# Endovascular treatment for acute M2 occlusion stroke within 6 hours-a retrospective real-world evidence

**DOI:** 10.3389/fcvm.2022.1063078

**Published:** 2023-01-10

**Authors:** Yi Xu, Wang Fu, Yongpeng Wang, Qianqian Bi, Qiwei Wang, Lu Yang, Quanbin Zhang, Feng Wang

**Affiliations:** ^1^Department of Neurosurgery, Shanghai Tenth People’s Hospital, Tongji University School of Medicine, Shanghai, China; ^2^Department of Neurology, Seventh People’s Hospital of Shanghai University of Traditional Chinese Medicine, Shanghai, China

**Keywords:** acute ischemic stroke, M2 occlusion, endovascular therapy, intravenous thrombolysis, stroke

## Abstract

**Background:**

We compared the efficacy and safety of endovascular therapy (EVT), intravenous (IV) thrombolysis and conservative treatment in M2 segment occlusion stroke based on a real-world database.

**Methods:**

We retrospectively analyzed the database of admitted patients with M2 segment occlusion between January 2018 and December 2020. The patients who were eligible for reperfusion treatment were assigned to EVT, IV thrombolysis or conservative treatment according to the exact management proceeding. The primary outcome was a score of 0 and 1 on the modified Rankin scale (mRS) at 90 days. The odds ratio (OR) for the primary outcome was adjusted for age, baseline National Institute of Health Stroke Scale score, and door-to-treatment time. The secondary outcomes were based on a mRS score from 0 to 2 at 90 days and the safety outcomes including symptomatic intracranial hemorrhage, and all-cause mortality. The data were analyzed by the logistical regression model, including baseline adjustments.

**Results:**

A total of 109 patients were included. Among them, 42 (38.5%) patients received EVT, 45 (42.5%) received IV thrombolysis and 22 (20.8%) received conservative treatment. The primary outcome based on a mRS score of 0 and 1, occurred in 66.7% of patients in the EVT group and 40% in the IV thrombolysis group (adjusted OR, 1.79; 95% confidence interval [CI], 1.19-2.68; *P* = 0.01). Symptomatic intracranial hemorrhage occurred in 1 patient (2.3%) in the EVT group and in 2 patients (4.4%) in the IV thrombolysis group (adjusted OR = 0.71, 95% CI: 0.13-4.07).

**Conclusion:**

EVT showed better functional outcomes than IV thrombolysis and conservative treatment in moderate to severe acute stoke patients with M2 occlusion. There was no significant difference in the three groups concerning the incidence of symptomatic intracranial hemorrhage.

## 1. Introduction

Based on the recent landmark trials, current guidelines recommend endovascular therapy (EVT) as the standard care for acute ischemic stroke with an occlusion of the distal internal carotid artery or proximal middle cerebral artery (1). These trials concluded that the combination of EVT with intravenous (IV) thrombolysis improves the 90-day outcomes of ischaemic stroke with internal carotid artery (ICA) or M1 segment of the middle cerebral artery (MCA) occlusion, within 6 hours from the stroke onset. However, occlusion of M2 segment has been underestimated in these trials and therefore the treatment of M2 segment remains controversial (2–4).

Previous study indicated that occlusion at a more distal MCA might be associated with unsuccessful recanalization (5). The PROMISE study compared the angiographic and clinical outcomes of aspiration thrombectomy in M1 and M2 occlusion, indicated that the M2 segment had similar outcomes to M1. These were represented by a comparable rate of recanalization, device-related severe adverse events, and post-treatment symptomatic intracranial hemorrhage (sICH) (6).

In addition, very few studies analyzed the different outcomes between EVT, IV treatment and conservative treatment in acute M2 segment occlusion. Only one sub-analysis of RESCUE-Japan Registry 2 compared the EVT and non-EVT treatment (including IV thrombolysis and conservative therapy). Although showing the same likelihood of sICH, the study showed that EVT might increase mortality (7).

In recent years, EVT and IV thrombolysis are increasingly being performed in our country. More acute stroke units tend to proceed with EVT for vessel occlusion but also for the middle-sized ones, particularly for the M2 segment. Whether EVT is safer and more beneficial than IV thrombolysis in treating M2 occlusion patients are becoming critical questions that need addressing. To support the clinical decision-making, this study aimed to investigate the efficacy and safety of EVT, IV thrombolysis and conservative therapy in patients with acute ischaemic stroke resulting from acute occlusion of the middle cerebral artery M2 segment. Meanwhile, we defined M2 segment according to Tomsick’s study (8).

## 2. Materials and methods

### 2.1. Study design and setting

This study is a multicentre, retrospective, cohort study in Shanghai, China. As a developed major city, Shanghai has more than 70,000 ischaemic stroke per year. There are more than 30 hospitals that provide 24/7 thrombolysis services, two of which (Tenth People’s Hospital and the Seventh People’s Hospital) are tertiary neurovascular centers in North and East Shanghai that are equipped to provide EVT. Both centers have a stroke team and an acute stroke unit that use a same acute stroke intervention protocol. The acute stroke patients are recognized by trained ambulance staff who informs the hospital-based triage nurse and acute stroke team before arriving at the emergency department (ED). On the patient’s arrival, the acute stroke team is ready to initiate the Code Stroke that consists in proceeding with the stroke imaging protocol including non-contrast CT brain, CT perfusion (CTP), CT angiogram (CTA) and informing the on-call interventional neuroradiologist to remotely access the stroke imaging and prepare for the EVT if required. By using the acute stroke protocol, the recorded average time of door-to-needle (from patients entering the hospital to the needle) was 41 min, and the average door-to-puncture (from patients entering the hospital to the puncture) was 78 min. We provide continuous stroke management from acute phase to post-discharge for up to 3 months. The collected data from these two metropolitan hospitals are from the same database.

### 2.2. Participants

The data were selected from the shared Stroke Database of the Tenth People’s Hospital and the Seventh People’s Hospital in Shanghai. An eligible dataset was included if the patient: (1) was recorded by the Stroke Database between 1st January 2018 and 31st December 2020; (2) was diagnosed with an acute ischaemic stroke resulting from M2 occlusion (the diagnostic selection criteria is as below); (3) received IV thrombolysis within 4.5 h or EVT within 6 h after onset; (4) had complete series of CT perfusion, CT angiogram or DSA images before treatment; (5) completed the follow-up review in 90 days post-stroke. Those who received bridged treatments, including EVT + IV thrombolysis were included in the EVT group. Those who were assessed to be eligible but refused to proceed with either EVT or IV thrombolysis treatment, were assigned to the conservative treatment group where they promptly received standard anti-platelet therapy. Meanwhile, patients with contraindication of thrombolysis or EVT would also receive conservative treatment.

Those who did not have analyzable images or did not complete the modified Rankin scale (mRS) assessment at 90 days after stroke, were excluded. This study was approved by the Tenth People’s Hospital Research Ethics Committee (approval number: 21k253) and the Seventh People’s Hospital Research Ethics Committee (approval number: 2021-B151). The informed consent was waived due to the retrospective nature.

In this study, we used the functional-anatomical M1-M2 classification to define the M2 occlusion. Arsing from M1, adjacent to ATA but larger or giving origin to ATA and distributing to the mid- and posterior temporal lobe were termed “posterior temporal M2 branch” and “holotemporal M2 branch”. The single vessel continuation of M1 beyond the posterior temporal or holotemporal M2 branches was termed “M2 trunk” (8).

It suggested that the filling of at least one M2 branch, including the existence of holotemporal artery or posterior temporal artery, was considered as M2 occlusion when using CTA, CTP with or without DSA. The imaging for each patient was reviewed by a neurologist and a neuroradiological specialist and a consensus opinion was reached to assert the presence of an M2 segment occlusion. Those without agreement were reviewed by an interventional neuroradiologist to determine a consensus.

### 2.3. Imaging protocol

According to the stroke care protocol at both hospitals, all patients with acute ischemic stroke received a multimodal CT assessment which included non-contrast CT brain, CTA and CTP, with or without a following DSA. Iodinated contrast (40-mL iohexol 350 mg/ml, Omnipaque 350; GE Healthcare, Milwaukee, WI, USA) was injected at 8 mL/s, and 40 images were acquired per second (total acquisition time, 44 s). The images were processed using a commercial version of RAPID automated software.

### 2.4. Interventions

Patients were managed according to the acute stroke treatment guideline and hospital acute stroke protocol of EVT and IV thrombolysis. When the patients were within the time window of 4.5 h, IV thrombolysis was performed. The IV thrombolysis combined with EVT was used when the CTP mismatched while the large vessel occlusion was diagnosed. On the other hand, when the patients were within the time window of 4.5-6 h, the EVT was used when the CTP mismatched while the large vessel occlusion was diagnosed. Or the patient would receive conservative treatment. Patients with contraindication of thrombolysis or EVT would also receive conservative treatment.

The performance of EVT was as follows: 8F or 6F guide tube was advanced at the end of C1 segment, then the micro wire (Boston Science, USA) was slowly advanced through the M2 occluded section, and the Navien catheter (EV3, USA) was advanced at the C4 segment over the micro wire. The micro wire was advanced across the occluded segment followed by the micro catheter (EV3, USA) which was confirmed to be in the true lumen by angiography. The Solitaire thrombectomy stent (Solitaire FR, Medtronic) was sent through the microcatheter and placed in distal true lumen of the occluded segment. After accurate positioning, the stent was released. After 5 minutes, the stent and microcatheter were withdrawn together for thrombectomy. The final angiography was performed to evaluate the lesions.

The IV thrombolysis was performed using tissue plasminogen activator at a concentration of 0.9 mg/kg and with a maximum of 90 mg. The patients received first a 10% of the total dose via intravenous bolus injection and then a continuous infusion within 1 h to finish the total dosage. The patient received retrieval therapy that may have been preceded by an IV t-PA. Conservative treatment was used for patients who were contraindicated to IV thrombolysis therapy and who failed to receive EVT for whatever reason.

### 2.5. Outcomes

The primary outcome was a score of 0 or 1 on the mRS at 90 days (indicating an excellent functional outcome with a return to all usual activities). Data were obtained by telephone or outpatient interview. The odds ratio (OR) for the primary outcome was adjusted for age, clinical severity of stroke (National Institute of Health Stroke Scale, [NIHSS] score) at baseline, and time from onset to treatment. The secondary outcomes were scored from 0 to 2 on the mRS at 90 days (indicating functional independence); the score (0 to 6) on the mRS at 90 days (with the distribution of scores in each trial group used in an ordinal analysis to assess functional improvement); safety assessment, including the sICH (defined according to the SITS-MOST definition (9) and all-cause mortality. An independent neurologist, who was blind to the treatment method, was responsible for the evaluation of mRS at 90 days by telephone consult or outpatient interview.

### 2.6. Data collection

Baseline characteristics included age, gender, vascular risk factors, pre-event mRS score, and NIHSS at presentation. In this study, the onset-to-CT time (measured in minutes) was used to represent the onset-to- ED (time from the onset to entering the ED) time due to a better accuracy of the CT recording time. The average time between ED-admission and CT performance was 38 min. Stroke onset time was evaluated according to the patients or the witness. For an unknown-onset stroke, the time when the patients were last noticed as unaffected, was considered to represent the onset.

### 2.7. Statistical analysis

The statistical analysis was performed using SPSS version 20.0 for Windows (IBM Co., Armonk, NY, USA). Continuous variables were presented as mean ± standard deviation (SD), and categorical variables were presented as frequencies and percentages. Fisher exact test was used to compare the difference at baseline between categorical variables, and Wilcoxon-Mann-Whitney test was used for continuous variables. The logistic regression model was used to compare the 90-day mRS, sICH, and mortality in the different treatment cohorts, that were adjusted by age, NIHSS score at baseline, and time from onset to treatment. In the analysis, EVT group was set as the reference, which was used to compare the IV group and conservative group. A *p*-value of less than 0.05 was considered statistically significant.

## 3. Results

Between the 1st of January 2018 and the 31st of December 2020, 2761 patients with acute ischemic stroke were screened, of which 109 had an M2-occlusion. The mean age of the study population was 69.4 ± 12.0 years, and 45.0% of them were females. All participants received the brain-scan within 6 hours after the onset. Forty-two (38.5%) participants underwent EVT, of which, 24 received thrombolysis beforehand (57.1% of all EVT cases), 45 received IV thrombolysis alone (41.3%), and 22 underwent conservative treatment (20.2%). The baseline characteristics of the three groups were detailed in Table 1. The conservative group, which was comprised of older patients, had a prolonged waiting time for CT scan in ED (door-to-CT time 54 min vs. 35.8 min for EVT and 32.5 min for thrombolysis). Although the conservative group had a relatively lower percentage of being well function at baseline (mRS ≤ 2: 77.2%), the baseline mRS did not show a statistical significance across the groups (*p* = 0.34). The patients who received EVT showed a longer time from emergency arrival to recanalization due to the time that was spent in preparation for the operation compared with IV thrombolysis (65 vs. 43 min). Otherwise, no significant difference was observed between EVT and IV thrombolysis at baseline.

**TABLE 1:** Baseline characteristics for study groups.

Characteristic	EVT (*n* = 42)	IV thrombolysis (*n* = 45)	Conservative treatment (*n* = 22)	*P*-value
Age — years (mean, SD)	67.4 (13.0)	68.7 (10.9)	74.71 (8.3)	0.046
Gender (Male) — N (%)	19 (45.2)	21 (46.7)	9 (40.9)	0.90
**Medical history — N (%)**				
Hypertension	29 (69.0)	34 (75.6)	16 (72.7)	0.79
Hyper-lipidemia	7 (16.7)	10 (22.2)	6 (27.3)	0.60
Diabetes Mellitus	10 (23.8)	12 (26.7)	4 (18.2)	0.75
Atrial fibrillation	14 (33.3)	18 (10.0)	12 (54.5)	0.26
Previous stroke/TIA	15 (35.7)	10 (22.2)	3 (13.6)	0.12
Ischaemic heart disease	9 (21.4)	10 (22.2)	4 (18.2)	0.93
Smoker (current)	11 (26.2)	14 (31.1)	6 (27.3)	0.87
Pre-onset mRS ≤ 2 — N (%)	38 (90.4)	39 (86.7)	17 (77.2)	0.34
Onset NIHSS (mean, SD)	13.1 (3.9)	11.7 (3.2)	13.1 (2.7)	0.07
Door to CT time (mean, SD)	35.8 (10.6)	32.5 (8.9)	54.0 (12.8)	<0.001
Door to puncture or needle time (Median, IQR)	65 (56-300)	43 (26-58)	NA	<0.001

EVT, endovascular treatment; IV, intravenous; tPA, tissue-type plasminogen activator; TIA, transient ischemic attack; NIHSS, National Institutes of Health Stroke Scale; mRS, modified Rankin scale. Door to CT time and door to puncture or needle time was calculated by minutes.

### 3.1. Efficacy

The primary outcome of the mRS score of 0 or 1 was attained by 66.7% of the patients in the EVT group, by 44.3% in the IV thrombolysis group and by 22.7% in the conservative treatment group. The adjusted OR of EVT to IV thrombolysis was 1.67, showing a significant difference between the groups (95% confidence interval [CI]: 1.10-2.53, *p* = 0.01). Given the door-to-puncture time being longer than that of the door-to-needle (65 min vs. 43 min), the adjusted OR became more significant by 1.79 (95% CI 1.19-2.68). Only 22.7% of the patients who received conservative treatment could achieve a mRS score of 0 or 1, which was significantly lower than that in the EVT group (adjusted OR: 3.04; 95% CI 1.37-6.74; *p* = 0.001) (Table 2).

**TABLE 2:** Multivariable logistic regression analysis for the 90-days follow-up mRS across three groups.

	EVT (*n* = 42) *N* (%)	IV-tPA (*n* = 45)				Conservative treatment (n = 22)			
		*N* (%)	*p*	OR (95% CI)		*N* (%)	*p*	OR (95% CI)	
				Unadjusted	Adjusted*			Unadjusted	Adjusted#
mRS ≤ 1	28 (66.7)	18 (40.0)	0.01	1.67 (1.10-2.53)	1.79 (1.19-2.68)	5 (22.7)	0.001	2.93 (1.32-6.52)	3.04 (1.37-6.74)
mRS ≤ 2	32 (76.2)	24 (53.3)	0.04	1.43 (1.04-1.97)	1.52 (1.11-2.07)	6 (27.2)	<0.001	2.79 (1.38-5.64)	2.84 (1.41-5.73)

OR, odds ratio. The OR was adjusted for age, onset NIHSS score, pre-onset mRS, hypertension, diabetes, hyper-lipidemia, current smoker, time from door to CT. *Comparison between EVT and IV-tPA; ^#^Comparison between EVT and conservative treatment.

**FIGURE 1:**
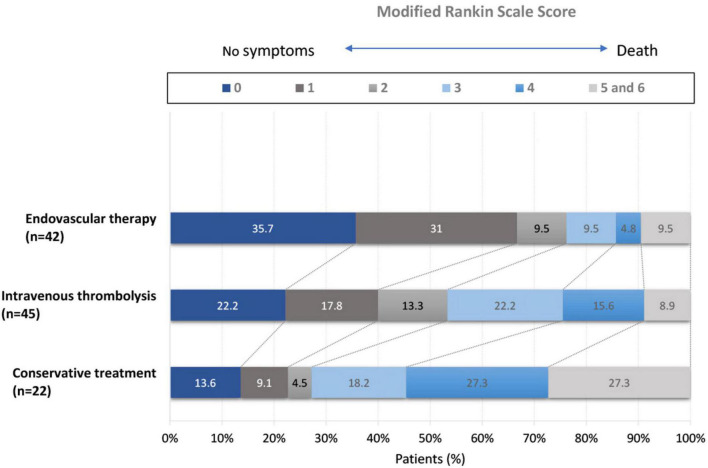
The mRS scores at 90 days among patients receiving endovascular therapy, intravenous thrombolysis, and conservative treatment.

Regarding the secondary outcomes, a mRS score of 0 to 2 was attained by 76.2% of the patients in the EVT group, 53.3% in the IV thrombolysis group (adjusted RR: 1.52; 95% CI 1.11-2.07; *p* = 0.04), and 27.2% in the conservative treatment group (adjusted OR: 2.84; 95% CI 1.41-5.73; *p* < 0.001) (Table 2). A shift analysis of the distribution of scores for each group, mRS did not show statistical significance between the groups’ difference in functional improvement at 90 days, due to the small sample size (Figure 1).

### 3.2. Safety assessment

The safety of the different treatments was assessed by sICH and mortality at 90 days. There was only one case in the EVT group and two cases in the IV thrombolysis cohort developed sICH. Given the small sample side, no statistical significances were observed (sICH 2.3 vs. 4.4%, *p* > 0.99; adjusted OR = 0.71; 95% CI: 0.13-4.07) (Table 3). No patients died in this study cohort.

**TABLE 3:** The safety assessment of the treatments: Symptomatic ICH and mortality.

	EVT (*n* = 42) *N* (%)	IV thrombolysis (*n* = 45) *N* (%)	*P*-value	OR (95% CI)	
				Unadjusted	Adjusted
Symptomatic ICH	1 (2.3)	2 (4.4)	>0.99	0.54 (0.05-5.69)	0.71 (0.13-4.07)

ICH, intracranial hemorrhage. The OR was adjusted for age, baseline NIHSS score and door-to-puncture or needle time by minutes.

## 4. Discussion

In this study, we retrospectively enrolled 109 patients. Among them, 42 (38.5%) patients received EVT, 45 (42.5%) received IV thrombolysis and 22 (20.8%) received conservative treatment. EVT showed its superiority in functional outcomes (attain of a mRS score ≤ 1) and safety compared with the other two kinds of treatments.

Due to the more distal position of M2, the tissue loss was conceptually smaller than M1 occlusion. Hence the potential improvement of functional status after treatment may be not significant as that in M1 (10). Furthermore, M2 segment branches were characterized by thinner vessel walls and a narrower lumen, making the procedure technically more difficult and the vessel wall more prone to perforation, increasing the risk of complications and early re-occlusion (11). Despite all this, previous studies found M2 occlusions involving the eloquent cortex may cause significant neurological impairment, and these patients may benefit from reperfusion therapy compared with medical therapy (12). Meanwhile, Rai et al found 56% of patients with M2 occlusions had poor clinical outcomes and a mortality rate of 27% (13). Lima et al concluded that the 6-month mortality similar to patients with M1 occlusions, indicating the necessity of treatment of M2 segment (14). Sarraj et al also found patients managed with ECR had significantly better 90-day clinical outcomes (62.8 vs. 35.4%, *P* = 0.001) and although the ECR group did show higher frequency of sICH compared to those treated by medicine (15).

Recent meta-analysis studies also demonstrated that mechanical thrombectomy in M2 segment may have similar outcomes in hemorrhagic transformation, functional independence and motility compared to M1 segment occlusion (16–18). However, the definition of M2 segment was various. The most common definition was the vertical portion of MCA in the Sylvian fissure, extending from the genu to the apex of the circular sclcus, which may lead to a disparity during treatment decision making. Tomsick et al proposed the functional-anatomical M1-M2 classification to define the M2 occlusion and concluded it was closely related to patient’s prognosis after endovascular therapy (8). In this study, we used the novel definition of M2 segment and compared the safety and clinical outcomes of EVT, IV thrombolysis and conservative treatment in M2 segment occlusion stroke based on a real-world database. This study provided real-world evidence of the beneficial superiority and non-of EVT safety compared with IV thrombolysis and conservative treatment among the patients with acute ischaemic stroke associated with M2 occlusion in two centers. The EVT indicated a significantly higher potential of post-stroke function maintenance at 90 days with a mRS ≤ 1 being 66.7% and a mRS ≤ 2 being 76.2%, while IV thrombolysis maintaining a mRS ≤ 1 by 44.3% and mRS ≤ 2 by 53.3%. In an ordinal analysis of the distribution of mRS scores at 90 days, the small sample size in each subscale did not provide sufficient power to detect the differences between the groups. Findings for the sICH showed no significant difference between EVT and IV thrombolysis in absolute number. Due to lack of deceased cases in the study cohorts, we were unable to conduct analysis for mortality.

In this study, the classification of M2 occlusion was different from that of previous studies. The lack of consensus brings challenges in selecting an appropriate M2-segment occlusion population for intervention. One of the more common methods to define the M2 segment relies on the use of anatomic boundaries, in which the M2 segment originates from the genu of the MCA as it takes a vertical turn in the Sylvian fissure. Another widely accepted theory adopted the branching pattern and identified post-bifurcation branches of MCA as M2 segment (19). Due to the high frequency of early bifurcation MCA anatomy, these two methods may obtain inconsistent results (20). In this study, we used a mixing strategy to differentiate M2 from M1 using the functional-anatomical method. It defines M2 occlusion as having at least one filling of the classic M2 branch. Unlike those definitions which rely on “bifurcation” as a key component, the functional-anatomical method reduces the confusion from anatomic variation and may reasonably include holo-temporal artery and posterior temporal artery, which extend out before MCA bifurcates as M2 branches.

Our results showed that the EVT group achieve a higher percentage in maintaining a mRS ≤ 0-2 at 90 days (73%), a lower sICH (2.3%) and mortality (no decease case) compared with the results of recent meta-analysis (mRS of 0-2: 59%, sICH: 4.9-10%, mortality: 7.7-16%) (8, 9). The success of EVT may be due to the selection criteria that used functional-anatomical M2 identification which was introduced in this study. The improvement of the EVT technique also escalated the benefit of post-stroke outcomes by successfully achieving a better recanalization and by shortening the door-to-treatment time.

However, this study had several limitations. Firstly, it is a retrospective cohort study that was conducted in a metropolitan city, where the selection bias needs to be considered. Meanwhile, patients in the EVT group may have higher NHISS scores and the retrospective design of the study with unknown confounding factors unable to be adjusted also had potential effect on the results. Secondly, the exclusion of patients by neuro-interventionists, due to the difficulty in clot retrieval performance may decrease adverse outcomes and mortality. Thirdly, some of the patients receiving EVT were refereed and transferred from other hospitals where they may have received intravenous treatment before admission. For those patients, it was difficult to track the initial treatment which may have an impact on the results. Finally, the small sample size increased type II error and reduced the significance of the results.

## 5. Conclusion

This study provided real-world evidence of the benefit and safety in EVT, IV thrombolysis and conservative treatment in moderate to severe acute stroke patients with M2 occlusion. EVT showed its superiority in functional outcomes and safety compared with the other two treatments. The results in this study may support the design of a treatment strategy for acute ischemic stroke with M2 occlusion. Additional large data analysis across different cities involving more hospitals, is required to broaden the results, especially in safety assessment.

## Data availability statement

The raw data supporting the conclusions of this article will be made available by the authors, without undue reservation.

## Ethics statement

The studies involving human participants were reviewed and approved by Tenth People’s Hospital Research Ethics Committee (approval number: 21k253) and the Seventh People’s Hospital Research Ethics Committee (approval number: 2021-B151). The patients/participants provided their written informed consent to participate in this study.

## Author contributions

YX and WF: conception, design, data collection, data analysis, and write and revise the manuscript. YW, QB, QW, and LY: conception, design, patient follow-up, literature review, and revise the manuscript. QZ and FW: conception, design, data analysis, result interruption, and revise the manuscript. All authors have final approval of the manuscript.
